# DINTD: Detection and Inference of Tandem Duplications From Short Sequencing Reads

**DOI:** 10.3389/fgene.2020.00924

**Published:** 2020-08-11

**Authors:** Jinxin Dong, Minyong Qi, Shaoqiang Wang, Xiguo Yuan

**Affiliations:** ^1^School of Computer Science and Technology, Xidian University, Xi’an, China; ^2^School of Computer Science and Technology, Liaocheng University, Liaocheng, China

**Keywords:** tandem duplications, DBSCAN, next-generation sequencing, read depth, mapping quality

## Abstract

Tandem duplication (TD) is an important type of structural variation (SV) in the human genome and has biological significance for human cancer evolution and tumor genesis. Accurate and reliable detection of TDs plays an important role in advancing early detection, diagnosis, and treatment of disease. The advent of next-generation sequencing technologies has made it possible for the study of TDs. However, detection is still challenging due to the uneven distribution of reads and the uncertain amplitude of TD regions. In this paper, we present a new method, DINTD (Detection and INference of Tandem Duplications), to detect and infer TDs using short sequencing reads. The major principle of the proposed method is that it first extracts read depth and mapping quality signals, then uses the DBSCAN (Density-Based Spatial Clustering of Applications with Noise) algorithm to find the possible TD regions. The total variation penalized least squares model is fitted with read depth and mapping quality signals to denoise signals. A 2D binary search tree is used to search the neighbor points effectively. To further identify the exact breakpoints of the TD regions, split-read signals are integrated into DINTD. The experimental results of DINTD on simulated data sets showed that DINTD can outperform other methods for sensitivity, precision, F1-score, and boundary bias. DINTD is further validated on real samples, and the experiment results indicate that it is consistent with other methods. This study indicates that DINTD can be used as an effective tool for detecting TDs.

## Introduction

Genome structural variations (SVs) are polymorphic rearrangements of 50 base pairs or greater in length and affect about 0.5% of the genome of a given individual (Eichler, 2012). SVs include deletions, insertions, duplications, inversions, and translocations of segments of DNA (Balachandran and Beck, 2020). Copy number variation (CNV) can be regarded as an important type of genome SVs (Redon et al., 2006; Chao and Tammi, 2009; Iacocca and Hegele, 2018). Tandem duplication (TD) is defined as a structure rearrangement whereby a segment of DNA is duplicated and inserted serially to the original segment (Olivier et al., 2003). Whole-genome sequencing (WGS) data from tumors have revealed that massive rearrangements, as in the tandem duplicator phenotype, are a specific cancer phenotype (Inaki and Liu, 2012). TDs commonly occur in some cancers (Stephens et al., 2009), particularly in ovarian and breast cancer genomes. A subset of ovarian cancer samples shares a marked TD phenotype with triple-negative breast cancers (Mcbride et al., 2012). The fms-like tyrosine kinase 3 internal TD (FLT3-ITD) is present in 30% of cases of acute myeloid leukemia (AML) (Kapoor et al., 2018). A novel recurrent TD in IFT140 was found in patients with uncharacterized ciliopathies using WGS (Geoffroy et al., 2018). Therefore, TDs play an important role in the mechanism of human disease, the detection of which has great significance for genome analysis and the study of human evolution.

Next-generation data has made it possible to detect and genotype SVs in the human genome. The primary strategies for the characterization of SVs include paired-end mapping (PEM), read depth (RD), split read (SR), *de novo* assembly, and a combination of the above strategies (CB). PEM uses discordant alignment features, such as insert size and directions of paired-end reads, to infer the presence of SVs (Korbel et al., 2007; Chen et al., 2009; Kai et al., 2009; Zeitouni et al., 2010). RD is based on the read counts aligned to genome windows (Yoon et al., 2009; Abyzov et al., 2011; Miller et al., 2011; Boeva et al., 2012; Yuan et al., 2018). If regions of some consecutive windows have a significantly higher or lower read count, they will be identified as CNV. SR uses the SR signals to infer SVs and their breakpoints. SR tries to align clipped reads and one-end-anchored reads to find the matching breakpoints or refine the breakpoints identified by discordant alignment reads (Jiang et al., 2012; Rausch et al., 2012; Zhang et al., 2012; Hart et al., 2013; Schroder et al., 2014; Wang et al., 2015; Guan and Sung, 2016). When the reads are aligned across breakpoints, they will be split into separate parts and only some parts will be mapped to the reference genome. The *de novo* assembly first arranges the contigs from the entire or unmapped sequencing reads, then aligns the contigs to the reference genome (Wang et al., 2011; Li, 2015; Zhuang and Weng, 2015; Kavak et al., 2017).

These strategies are commonly used in detecting SVs, but they all have certain defects. RD can only detect unbalanced SVs, and the boundaries of the regions it detects are often rough. SR can detect SVs at the nucleotide level but there are usually a lot of discordant alignments, and thus SR is often integrated with other strategies. The strategy of *de novo* assembly requires assembling short reads, which has high time and space challenges. CB integrates some or all of the above strategies (Jiang et al., 2012; Layer et al., 2014; Bartenhagen and Dugas, 2016; Chen et al., 2016; Eisfeldt et al., 2017; Soylev et al., 2019), and often works more effectively than strategies using merely one signal.

In this work, we focus on the detection of TD regions and inference of their breakpoints from short sequencing reads. We first provide a brief introduction to existing methods that are used to detect TDs. VNTRseek (Gelfand et al., 2014) maps short sequencing reads to a set of reference TDs and then identifies putative TDs based on the discrepancy between the copy number of a reference and its mapped read; it is designed for minisatellite TDs. When the TD length is medium or long, it does not work well. TARDIS (Soylev et al., 2019) detects TDs by calculating maximal valid clusters for SVs that encompass all the valid read pairs and SRs for the particular SV. If discordant read pairs and SRs are mapped in special opposing strands, they are identified as TDs. DBDB (Yavas et al., 2014) predicts TDs based on the distribution of fragment length using discordantly aligned reads, and the breakpoints are inferred by applying a probabilistic framework that incorporates the empirical fragment length distribution to score each feasible breakpoint. LUMPY (Layer et al., 2014) is also based upon a general probabilistic representation of an SV breakpoint. TIDDIT (Eisfeldt et al., 2017) utilizes discordant read pairs and SRs to detect the location of SVs with the RD signal for classification. These methods all have assumptions. RD-based methods often assume observed RD and that the number of discordant read pairs follows a Poisson distribution. PEM-based methods assume the insert size follows a normal distribution. Some derive general distributions or a probabilistic framework from empirical fragment length distributions. But the real distributions of the observed RD signals are uncertain due to sequencing error, mapping error, GC content bias, and uneven nature of the data, thus deviating from the assumed distribution.

We propose a new method called DINTD (Detection and INference of TDs) using density-based spatial clustering of applications with a noise (DBSCAN) algorithm (Ester, 1996; Schubert et al., 2017). DINTD builds a pipeline that integrates the RD and SR signals mentioned previously. Also, we integrate mapping quality (MQ) signals (Li et al., 2008), which is a measure of the confidence that a read comes from the position it is aligned to by the mapping algorithm. In the first stage of the pipeline, the rough TD regions will be detected. To achieve this goal, RD and MQ signals are pre-processed and are treated as two features of the DBSCAN algorithm. To smooth consecutive bins, the TV (total variation) model (Duan et al., 2013) is used. The running result of DBSCAN provides clusters for the bins and TD regions are detected as noise. The distances between bins are frequently calculated when the DBSCAN algorithm is searching for the nearest neighbors. To reduce the required number of distance calculations, the 2D binary search tree (BST) approach (Bentley, 1975) is used to divide the search space into nested orthotropic regions. In the second stage of the pipeline, the boundaries of TD regions are refined based on the discordant SR signals. We test the performance of DINTD on simulation data by comparing it to existing methods. The experiment results demonstrate that DINTD achieves superior results in terms of sensitivity, precision, F1-measure, and boundary bias. We further apply DINTD to real sequencing samples to demonstrate its reliability.

## Methods

### Workflow of DINTD

**FIGURE 1:**
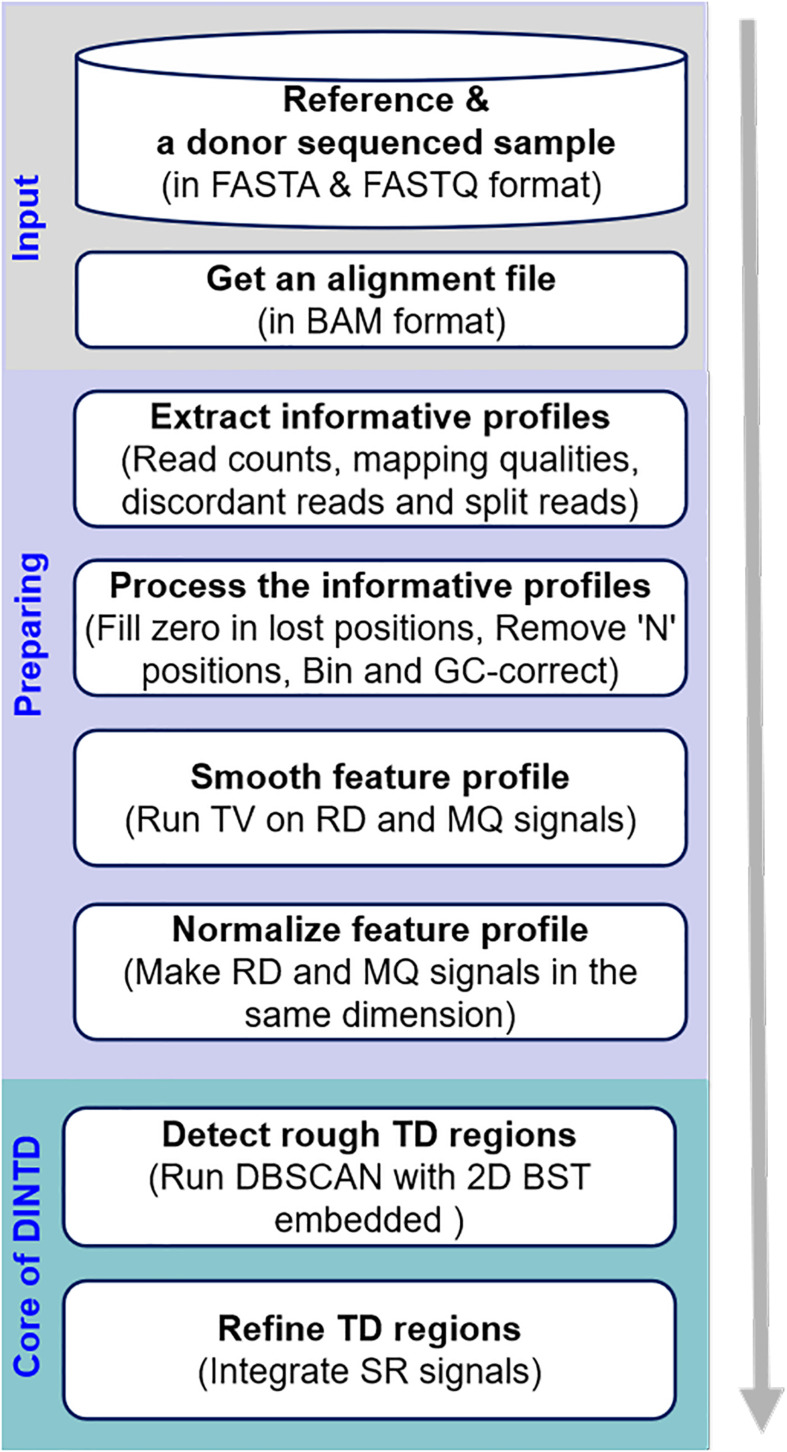
Diagram showing the workflow of the DINTD method. DINTD is composed of three primary parts, including input files, preparing informative profiles, and declaring TDs.

The workflow of the DINTD method is depicted in Figure 1. It consists of three main steps. In the first step, a donor sample and a reference genome (e.g., GRCh38) are prepared as the input. An alignment file (in BAM format) is obtained by aligning all the short reads to the reference genome utilizing the BWA-MEM approach (Li and Durbin, 2009). BWA is one of the most popular alignment tools due to its high accuracy. The alignment file is sorted by the genome position utilizing SAMTools software (Li et al., 2009). SAMTools is a popular library that provides utilities for manipulating high-throughput sequence alignments. In the second step, read counts and mapping qualities are extracted from the alignment file and put into the feature profile. SRs and discordant reads are extracted and put into the split profile. A pre-processing of the feature profile can then be carried out, such as dealing with value-lost and N positions to generate bins and correcting GC-bias for bins across the genome. In this step, the entire genome is divided into a number of continuous and non-overlapping bins; two features, including RD and MQ signals, can be obtained. These two features can be smoothed using the TV model to reduce noise. In the third step, a pipeline composed of detection and refinement is used to detect TD. The pipeline integrates the RD- and SR-based strategies. Some bins are detected as noise using DBSCAN based on RD and MQ signals. To speed the search for the nearest neighbor, the 2D BST strategy is embedded in the DBSCAN algorithm. For the detected noised bins, we merge the continuous ones into a large segment and that is then regarded as a rough TD region. Subsequently, we further use the split profile and the breakpoint positions of the TD regions are inferred. In the following subsections, the principle and implementation of each step will be described in detail.

### Pre-processing of Informative Profiles

The data pre-processing of informative profiles provides a data foundation for the pipeline of detecting TDs. It includes N position processing, RD and MQ calculation, GC correction, noise smoothing, and normalization.

#### Processing of N Positions

In any version of a reference genome, there are a large number of N values in genome positions (Yuan et al., 2019). The value of N means that the base has not been determined yet in the construction of the reference genome. The short reads are composed of A, T, C, and G. When a short read is compared with an N on the reference genome, the read count will be equal to zero. If the regions of N are removed directly, the final results about tandem duplication positions are biased. The observed read count is biased from the real read count since some reads have not mapped to the reference due to the N positions. To solve this problem, N positions in the reference genome are saved. The read count at each N position can be set to NA to represent the uncertain data. The read count of non-N positions is measured by counting the number of mapped reads.

#### Calculation of RD and MQ

The read count profile can be divided into non-overlapping bins. The *RD* for each bin on the reference genome can be calculated using the following formula:

(1) $$R\,D_{i}=\frac{\sum_{j=1}^{l\,e\,n\,\_\,b\,i\,n_{i}}R\,C_{j}}{l\,e\,n\,\_\,b\,i\,n_{i}}.$$

*RD*_*i*_ and *RC*_*j*_ represent the *RD* value and the *RC* value of the *j*-th position for the *i*-th bin, respectively, and *len_bin_*i*_* represents the length of the *i*-th bin, which is specified by the user, such as 2000 bp. If the *RD* value of a bin is equal to NA, this bin will be filtered out.

The calculation of *RD* is slightly different from traditional approaches (Yoon et al., 2009) that assign each read only once with its start position. However, if a read matches the breakpoint of two adjacent bins of the reference genome, the traditional method only increases the *RD* value of the previous bin by one, whereas our calculation method can increase the *RD* value of both bins.

The *MQ* value for each bin on the reference genome can be calculated using formula (2). It is similar to the calculation method of *RD*, except that the read count is modified to the value of *MQ*. The value of *MQ* during the alignment can be directly extracted from the alignment file.

(2) $$M\,Q_{i}=\frac{\sum_{j=1}^{l\,e\,n\,\_\,b\,i\,n_{i}}M\,q_{j}}{l\,e\,n\,\_\,b\,i\,n_{i}}.$$

*MQ*_*i*_ represents the value of *MQ* for the *i*-th bin and *Mq*_*j*_ represents the value of *MQ* of the *j*-th position for the *i*-th bin.

#### Correction of GC Content Bias

Sequence coverage on the Illumina Genome Analyzer platform is influenced by GC content (Bentley et al., 2008; Dohm et al., 2008). Therefore, we need to adjust the value of *RD* and *MQ* for each bin based on the observed deviation in coverage for a given G and C percentage (Dohm et al., 2008; Yoon et al., 2009; Abyzov et al., 2011; Boeva et al., 2012). The adjustment can be calculated using the following formula:

(3) ri∼i=nnG⁢C⁢ri.

In this, $\tilde{r_{i}}$ and *r*_*i*_ represent the corrected and original value of *RD* or *MQ* of the *i-*th bin, respectively, *n* represents the whole median of all the bins; and**n*_*GC*_* represents the median *RD* or *MQ* of all bins that have a similar G and C percentage as the *i-*th bin. The similar GC percentage is defined as the bins whose GC percentage deviation from the GC percentage of the *i*-th bin does not exceed 0.001.

#### Denoising Using TV (Total Variation)

Due to the noise data during the sequencing process, the *RD* and *MQ* between adjacent bins may vary randomly. The *RD* and *MQ* between adjacent bins have a natural correlation (Yuan et al., 2012, 2019), so the *RD* and *MQ* after GC corrections need to be further smoothed and denoised. Traditional median denoising and linear denoising do not distinguish between edges and noise. TV is based on the principle that signals with excessive (and possibly spurious) detail have high total variation, which is only sensitive to noise and can preserve edge information between bins. So, the TV method can be used to denoise *RD* and *MQ*. *RD* and *MQ* can be smoothed using the following formula:

(4) $$\underset{a}{m\,i\,n}\left\{\frac{1}{2}\,{||\text{b-a}||}^{2}+\lambda \,{||D\,a||}_{1}\right\}.$$

In this, *a* and *b* represent the vector forms of *a*_*i*_ and *b*_*i*_, i.e., *a* = [*a_1_,a_2_*,…,*a*_*n*_]^T^ and *b* = [*b*_1_,*b*_2_,…,*b*_*n*_]^T^; *n* is the number of bins; T represents the transpose operation; *a* represents the denoised signal; *b* represents the signal obtained from the above step; λ is a penalty parameter; ||b−*a*||^2^ denotes the L2 norm, i.e., the Euclidean distance between *a* and *b*; and ||.|_|1_ denotes the L1 norm, i.e., the Manhattan distance. *D* is a matrix of the size (*n*−1)×*n* that calculates the first-order derivatives of signal *a*:

(5) $$D=\left[\begin{array}{ccccccc} -1 & 1 & 0 & . & . & . & 0\\ 0 & -1 & 1 & 0 & . & . & .\\ . & . & . & . & & . & .\\ . & & . & . & . & & .\\ . & & & . & . & . & 0\\ 0 & . & . & . & 0 & -1 & 1 \end{array}\right].$$

The symbol λ represents the penalty parameter that controls the trade-off between the first term (which can be called fitting error) and the second term (which can be called the total variation penalty). It is difficult to determine the value of λ (Condat, 2013; Duan et al., 2013; Yuan et al., 2019). When it tends to zero, the effect of the penalty term is minimal, and *a* is equal to *b*. When it tends to positive infinity, the effect of the fitting error is minimal and the denoised signals are all equal. Our method allows the user to specify the value of the parameter λ.

#### Normalization

*RD* and *MQ* will serve as two features of the DBSCAN algorithm. *RD* is the average number of short reads aligned to each position in a bin and *MQ* is the average *MQ* of short reads aligned to each position of a bin. The value of *MQ* is much greater than the value of *RD*. When the DBSCAN algorithm looks for the nearest neighbor, a distance calculation equation is needed. Then *MQ* will affect the search for the nearest neighbor and the influence of *RD* becomes smaller. To reduce the influence of value ranges of different features on the DBSCAN algorithm, *MQ* can be normalized using the following formula:

(6) $$\overset{\text{∼}}{M\,Q}=\frac{M\,Q-M\,Q_{\min}}{M\,Q_{\max}-M\,Q_{\min}}\,\left(R\,D_{\max}-R\,D_{\min}\right)+R\,D_{\min}.$$

The value of *MQ* is transformed by scaling to the range of the *RD* value. This normalization method will not change the data distribution. Then the distance calculation is meaningful and the convergence rate of the gradient descent algorithm is faster.

### Detection of Rough TDs

In this step, we focus on the detection of rough TDs. The implementation is based on the principle that RD and MQ signals in the TD regions are different from other regions where no mutation has occurred. Here, MQ is used as a feature of the method. MQ describes the reliability of read alignment to a position in the reference sequence, which equals −10log10p(x). Here, p(x) is an estimate of the probability that the alignment position is wrong. It can be combined with other features for variation detection (Zhao et al., 2020). If there are TDs in the genome, then a read is mapped to multiple positions. In the BWA-MEM algorithm (Li and Durbin, 2009), the best one is selected; and if there are two or more best-matching positions, one is randomly selected from them. But according to a previous calculation method (Li et al., 2008), the value of MQ will still be relatively low. If we assume a TD in the genome of interest, MQ is not the intrinsic feature. From the perspective of observed sequencing reads without knowing where TDs occur, read mapping can provide much information for finding TDs. Here, RD is chosen as the measurement for evaluating whether each genome region is different from others, and then making a declaration for TDs accordingly. Since MQ can influence the calculation of RDs and it is not easy to eliminate the influence via a cutoff value, we use such factors together with RD as part of the features for the detection of TDs. If low quality reads simply are filtered at the onset, the depth of coverage is equivalent to a reduction. The depth of coverage of the genome is close to average, so we do not filter the low quality reads out.

If the pre-processed RD and MQ features are considered as points of the 2D data space *S*, then the difference of these two signals between different regions can be regarded as the difference of density between the regions. The core idea of DBSCAN is that for a given radius and minimum number of data points, the neighborhood of each point in a cluster has to contain at least a minimum number of points (Ester, 1996; Schubert et al., 2017). Furthermore, the density within the areas of noise is lower than the density in any of the clusters. This is suitable for our goal of detecting TD regions, which can be viewed as noise containing lower density. Therefore, it is meaningful to use DBSCAN for the detection of TD regions. For simplicity of description, the two features (*RD*_*i*_, *MQ*_*i*_) of the *i-*th bin can be called a point *o*_*i*_ in space *S*. Before introducing the algorithm, several related definitions will be introduced, i.e., the ε-neighborhood of a point *o*, core point, border point, directly density-reachable, density-reachable, density-connected, cluster, and noise (Ester, 1996; Schubert et al., 2017).

Definition 1: The ε- neighborhood of a point *o* is defined by the following formula:

(7) $$N_{\varepsilon }\,\left(o\right)=\left\{q\in S|d\,i\,s\,t\,\left(o,q\right)\leq \varepsilon \right\},$$

where *dist*(*o*,*q*) represents the distance function between *o* and *q*. The function *dist* works with any distance function, such as Manhattan distance, or Euclidean distance. Here we use Euclidean distance.

Definition 2: If the ε- neighborhood of a point *o* contains at least *MinPts* points, then *o* is called the core point. It is defined as the following:

(8) $$\left|N_{\varepsilon }\,\left(o\right)\right|\geq M\,i\,n\,P\,t\,s.$$

|*N*_ε_(*o*)| represents the number of points in *N*_ε_(*o*).

Definition 3: If *o* is a non-core point and is in the ε- neighborhood of a certain core point, then *o* is called a border point. There are core points in the ε- neighborhood of *o*. It is defined as the following:

(9) $$q\in N_{\varepsilon }\,\left(o\right)\,⋂S_{c}.$$

*N*_ε_(*o*) represents the ε- neighborhood of *o*, and *S*_*c*_ represents the set of core points. The set of non-core points can be represented by *S*_*nc*_ = *S*\*S*_*c*_.

Definition 4: If a point *q* is in the ε-neighborhood of a point *o*, and *o* is the core point, then *q* is directly density-reachable from *o*. It is defined by the following formula:

(10) $$q\in N_{\varepsilon }\,\left(o\right)\,a\,n\,d\,\left|N_{\varepsilon }\,\left(o\right)\right|\geq M\,i\,n\,P\,t\,s.$$

Definition 5: If there is a chain of points {*p*_1_,*p*_2_,…,*p*_*n*_}, and *o* = *p*_1_,*q* = *p*_*n*_, then *q* is density-reachable from *o.* Here *p*_*i*_ is directly density-reachable from point *p*_*i–1*_.

Definition 6: If a point *p* is density-reachable form a point *o*, and a point *q* is density-reachable from point *o* too, then point *p* is density-connected to point *q*.

Definition 7: If a non-empty subset *C* of space *S* satisfies Maximality and Connectivity, then *C* is called a cluster. Maximality is achieved when *o* ∈ *C* and *q* is density-reachable from *o*, and then *q* ∈ *C*. Connectivity is achieved when *o* ∈ *C* and *q* ∈ *C*, and then *o* is density-connected to *q.* Here *o* and *q* are random points in the space *S*.

Definition 8: The noise is a set of points not belonging to any cluster. It can be defined by the following formula:

(11) $$S_{n\,o\,i}=\left\{o\in S|\forall i:o\notin C_{i}\right\}.$$

Here, *o* is a data point and *C_i_* is a cluster of the space *S*. The set of noise points can be represented by *S_noi_* = *S*\(*S_c_*⋃*S_nc_*).

For a given ε and *MinPts*, Algorithm 1 describes the steps of DBSCAN in detecting TD bins.

**ALGORITHM 1:** Detecting TD bins.

1: Retrieve all data points to find *S*_*c*_;
2: Choose an arbitrary point *o* in *S*_*c*_ and retrieve all points density-reachable from point *o.* A cluster *C*_*o*_ is generated;
3: Remove the points in *C*_*o*_ from the remaining *S*_*c*_;
4: Repeat steps 2 and 3 from the updated *S*_*c*_ until all the core points are retrieved or removed.

In step 2, to obtain all points density-reachable from core points, one method is an exhaustive search, which sequentially calculates the distance from each point to the core point, and then takes the *MinPts* points with the smallest distance. This method is a naive nearest neighbor search. In the naive nearest neighbor search, a large number of distance calculations are needed. To reduce computational cost and speed up the search for density-reachable points, the strategy of the binary search tree can be used if there is only one feature *RD*. But we use two features *RD* and *MQ*, so 2D BST can be embedded in DBSCAN.

The 2D BST is a binary tree structure which recursively partitions the parameter space along the data axes, dividing it into nested orthotropic regions into which data points are filed (Bentley, 1975). The 2D BST has the properties of a binary search tree. For example, if its left sub-tree is not empty, the values of all nodes in the left sub-tree are less than the values of its root nodes; if its right sub-tree is not empty, the values of all nodes in the right sub-tree are greater than the value of its root node; its left and right sub-trees are binary search trees too. Algorithm 2 describes the steps of tree building.

**ALGORITHM 2:** Building 2D BST.

1: Select a feature, and then select the median *m* of this feature as a pivot to divide the data point space *S* to obtain two sub-collections;
2: Create a tree node to store (*RD*, *MD*) corresponding to *m*;
3: Repeat steps 1 and 2 for two sub-collections until all sub-collections can no longer be divided. If a sub-collection can no longer be divided, save the data in the sub-collection in the leaf node.

In step 2, the MD feature is selected for the first time and the RD feature is selected for the next iteration. These two features are used alternately to divide the space *S*. The following search process is also based on this feature order. Algorithm 3 describes the method of finding the density-reachable points within a distance of ε from a point *o*.

**ALGORITHM 3:** Finding the density-reachable points.

1: Compare the value of the split dimension of *o* and the split node, enter the left sub-tree if the value of the split dimension of *o* is less than or equal that of the split node, otherwise enter the right sub-tree;
2: Repeat step 1 until the leaf node, which is in the same subspace as *o* and is the approximate nearest neighbor of *o*;
3: Backtrack the search path and determine whether there are other sub-spaces of the node. If there is a point whose distance from *o* is less than ε, jump to other sub-space to search and add it to the search path;
4: Repeat step 3 until the search path is empty.

After performing the DBSCAN and 2D BST algorithms, the noise points returned can then be regarded as the set of bins where TD occurs. So, we can connect the consecutive bins to get the TD regions. This method uses the RD and MQ signals of the bin, so the region boundaries are not accurate. Subsequently, we will use the SR signals to refine the rough TD regions.

### Inference of Precise TD Region

With the rough TDs detected, we further infer the precise boundaries of the TD regions based on the SR signals. Generally, in the alignment result of short sequencing reads to the reference genome, most of the aligned reads are completely concordant. As for the discordant alignment states, we consider two situations: one is to skip first and then match. We call this case the post-alignment, and can be denoted as *x* “S”(*L*-*x*) “M.” Here *x* is the number of mismatched bases; *L* is the length of the short sequencing read; “S” is the clip on the sequence and “S” can be a soft clip or hard clip in the BWA-MEM algorithm. “M” is defined as match. The other case is to match first and then skip. We call this case pre-alignment, and can be denoted as *y* “M” (*L*-*y*) “S.” Here *y* is the number of matched bases. In aligning, in addition to the marked characters of “M” and “S,” there are other marked characters. Our method is to focus on detecting TDs, so we will not consider other marked characters.

**FIGURE 2:**
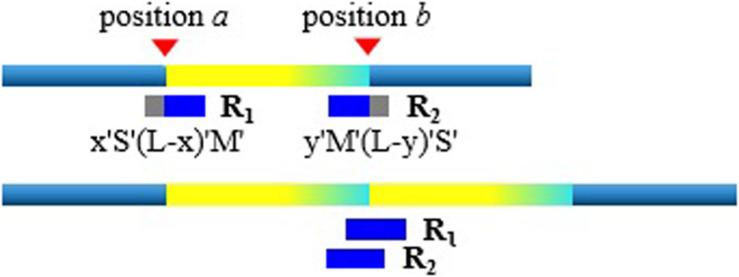
An example illustrating the pre-alignment and post-alignment approaches.

When the short sequencing read spanning the breakpoint aligns to the reference genome, there will be discordance of pre-alignment or post-alignment. An example is shown in Figure 2. When the short sequencing read *R1* aligns to the reference genome, it may match to the position *a* or the position *b*. The positions of *a* and *b* are the boundaries of the TD region. The BWA-MEM algorithm randomly selects a reference genome position for this short sequencing read of multiple matching. If *R1* matches near the position *a*, this is the post-alignment, where the low coordinate position of the TD region can be determined by *a* = *R*1.*pos*. Here *R1*.*pos* represents the position of the reference genome matched by *R1*, which can be directly extracted from the BAM file. To explain the mismatch more clearly, we give the other matching example of the short sequencing read *R2*. Assuming that it matches near the position *b*, that is, there is a pre-alignment, then the high coordinate position of the TD region can be determined by *b* = *R*2.*pos* + *y*−1. Here *y* represents the number of bases that are matched.

There are probably many discordant alignments, but not all mismatches can be used to determine breakpoints. We have detected the rough TD regions, which can be denoted as [*a*, *b*], using DBSCAN and 2D BST. So, we can now search for the discordant alignment near the TD region boundary, denoted as [*a-*, *b+*]. Thus, precise positions of the TD region boundary are inferred. It can improve the TD boundary accuracy to the nucleotide level, rather than the bin level. Algorithm 4 describes the method of inferring the precise TD region.

**ALGORITHM 4:** Inferring the precise TD region.

1: Scan all TD regions, represented as [a, b];
2: For each [*a*_*i*_, *b*_*i*_], extract all discordant alignment within the range of [*a*_*i*_-, *b*_*i*_+];
3: If there are post-alignments, modify *a* according to the post-alignment boundary changing method; If there are pre-alignments, modify *b* according to the pre-alignment boundary changing method;
4: Repeat steps 2 and 3 until all TD regions have been processed.

## Results

The DINTD software is implemented in Python language based on the methods described above, and the code is publicly available at https://github.com/SVanalysis/DINTD. The software is easy to install and requires a BAM file sorted by coordinate as input.

To evaluate the performance of DINTD, we conduct experiments by using simulation data first. This is because simulation data can provide ground truths for us to quantify sensitivity and precision (Yuan et al., 2017). From the experiment results, we compare metrics such as sensitivity, precision, F1-score, and boundary bias with existing methods (Rausch et al., 2012; Eisfeldt et al., 2017; Soylev et al., 2019). DINTD is run on real short sequencing data obtained from the 1000 Genomes project (Genomes Project et al., 2015; Sudmant et al., 2015) and EGA^1^. Since there is no single answer in real samples, the overlapping density score (Yuan et al., 2018) for the results among the methods is analyzed to show the reliability of DINTD. During the experiments, the parameter related to *RD* and *MQ* calculation is set to *len_bin* = 2000. The parameter related to TV denoising is assigned by users. By default, it is set to λ = 0.25. *MinPts* set as twice the number of features is appropriate (Schubert et al., 2017). So, in our algorithm, *MinPts = 4.* Users assign a value to the parameter *ε.* By default, it is set to *ε = 0.7.* Also, different values of parameters *len_bin* and λ will impact the results. A detailed discussion is provided in Supplementary Text.

### Simulation Studies

The comprehensive software SInC (Pattnaik et al., 2014) and seqtk^2^ are used to generate various short sequencing data sets based on chromosome 21 in the reference hg19. Here the reference genome can also be hg38. The sequence coverage is set to 10X, 20X, and 30X, and the tumor purity is set 0.3–0.9. In each configuration, 50 replicated samples are generated. For each simulation replication, a total of 10 TD regions are embedded, and the number of duplications changes from 1 to 6. The number of bases in the TD region is from 10,000 to 50,000.

**FIGURE 3:**
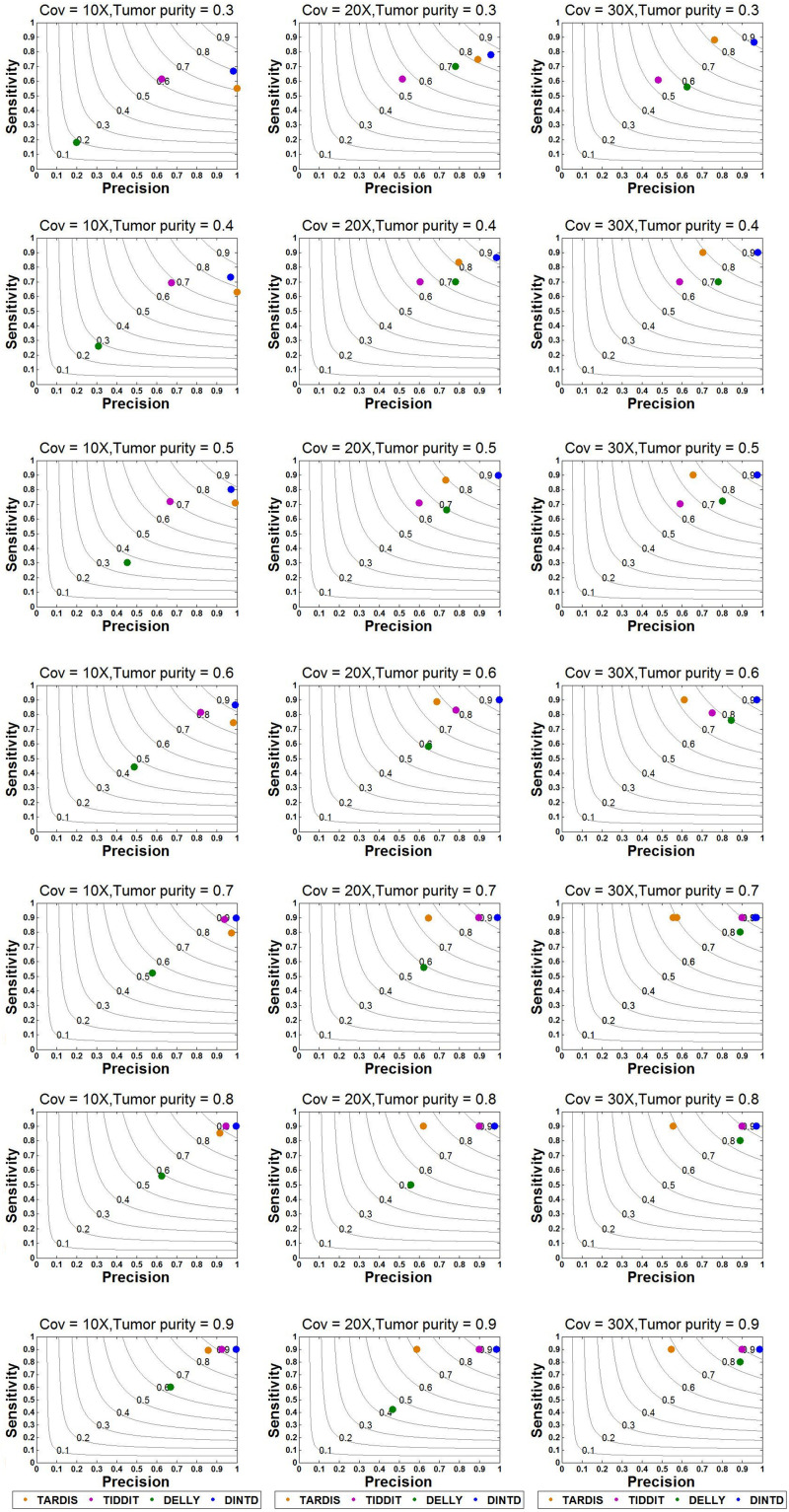
Sensitivity and precision between DINTD and three other methods (TARDIS, TIDDIT, and DELLY) when the sequence coverage is 10X, 20X, and 30X. F1-score levels are compared and shown by the gray curves.

Based on this simulation dataset, DINTD and the other three methods are performed. For the evaluation, metrics such as sensitivity, precision, F1-score, and boundary bias are used. The running times of these methods are also evaluated, and the result of comparison is showed in Supplementary Text. Sensitivity is defined as the ratio of true positives to true positives and false negatives, which is the ratio of the number of true TDs to the total TDs in the donor genome. Precision is defined as the ratio of true positives to true positives and false positives, which is the ratio of the number of true TDs to the total TDs detected by the method. Here, if half of the region of one real TD is covered by one of the regions of the called TDs, one true positive is counted. The overlapping intervals are half of the region of one real TD. Taking into account the sensitivity and precision, the F1-score can be regarded as a harmonized average of sensitivity and precision, and it is defined as 2 times the product of sensitivity and precision divided by the sum of sensitivity and precision. The boundary bias is defined as the deviation of the detected TD boundary from the actual TD boundary at the nucleotide level. To demonstrate the stability of the algorithm performance, we calculate each mean of different evaluation metrics in 50 samples for each sequence coverage and purity configuration. The results of sensitivity, precision, and F1-score calculations are presented in Figure 3.

**FIGURE 4:**
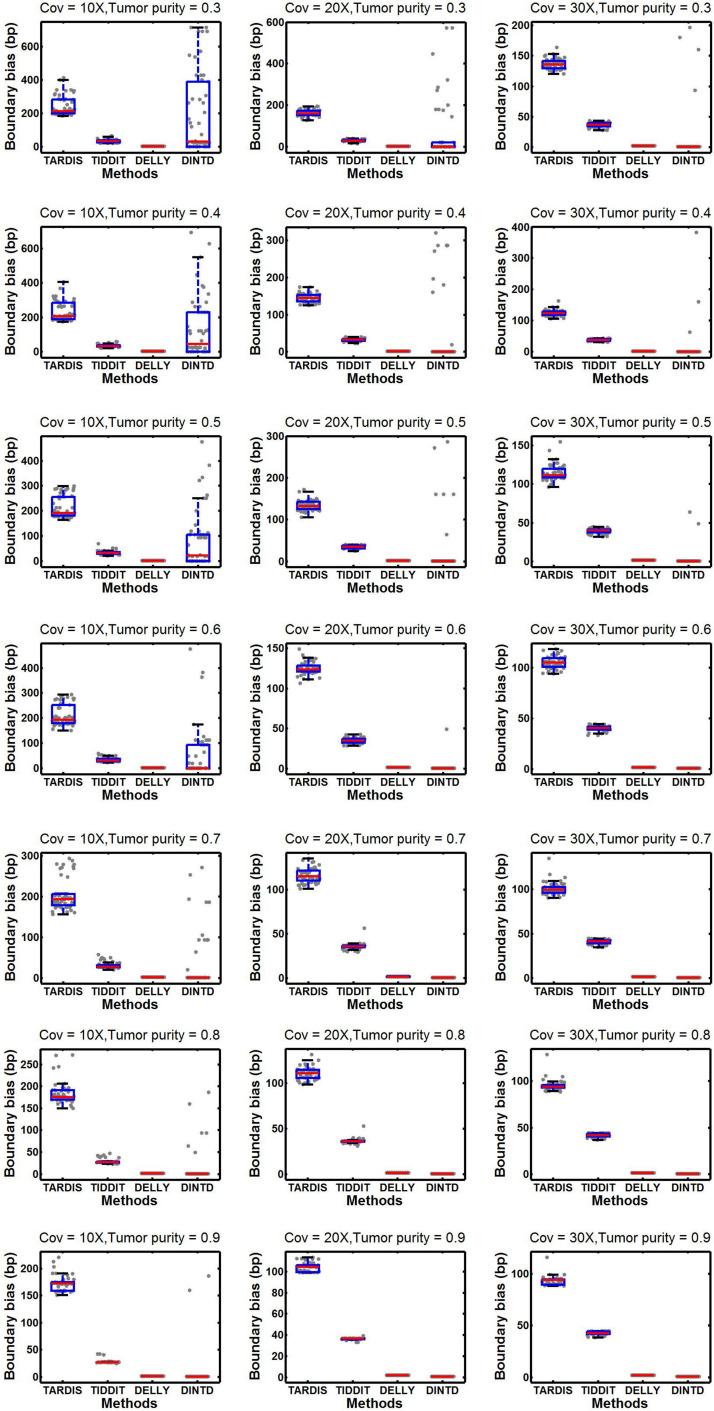
Comparisons of boxplot of the boundary bias between DINTD and three other methods (TARDIS, TIDDIT, and DELLY) when the sequence coverage is 10X, 20X, and 30X. To better demonstrate the distribution of data, we draw boundary biases of 50 experiments under each configuration uniformly with gray dots under each method.

According to the comparisons, for most algorithms, as the sequencing depth increases, the value of the F1-score and sensitivity increase slightly whereas precision decreases slightly. DINTD achieves the highest F1-score at all different sequence coverage and purity configurations. When the sequence coverage is at 10X, in terms of F1-score, DINTD is followed by TIDDIT, TARDIS, and DELLY when the purity is higher; and followed by TARDIS, TIDDIT, and DELLY when the purity is lower. In terms of sensitivity, DINTD, TARDIS, and TIDDIT are similar, with DELLY the lowest. In terms of precision, when the purity is higher, DINTD is the best, followed by TIDDIT, TARDIS, and DELLY; when the purity is lower, TARDIS is the best. When the sequence coverage is at 20X, in terms of F1-score, TIDDIT is the better if the purity is higher and TARDIS is better if the purity is lower. In terms of sensitivity, when the purity is higher, DINTD, TARDIS, and TIDDIT have similar performance, with DELLY the lowest; when the purity is lower, the performance of DINTD is the best, followed by TARDIS, TIDDIT, and DELLY. In terms of precision, DINTD has the best performance, followed by TIDDIT, DELLY, and TARDIS when the purity is higher, and followed by TARDIS, TIDDIT, and DELLY when the purity is lower. DELLY does not seem to perform well on the sensitivity, precision, and F1-score metrics. But when comparing the boundary bias, it does perform well. The smaller the boundary bias, the higher the accuracy of the method. The boxplot of boundary bias for each method is shown in Figure 4.

From Figure 4, we can see that as the purity and sequencing coverage increase, the boundary bias decreases. When the sequencing coverage is at 10X and the tumor purity is relatively low, DELLY performs best. When the tumor purity increases, the performance of DINTD improves so that it is slightly better than DELLY. When the sequencing coverage is at 30X, DINTD is always better than DELLY. These two methods are followed by TIDDIT and TARDIS.

We performed statistical tests to calculate a *P*-value for each pair of results (i.e., the result of DINTD and that of each of other methods). The results are provided in Supplementary Text. The central point is to test the difference between each pair of samples. For example, in our experiments with 10 simulated TDs, each method has obtained 10 values to reflect the boundary bias, and then our purpose is to test the difference between two samples each with 10 values. Here, we adopt the permutation test methodology. The idea is to choose a statistic and generate a number (e.g., 10,000) of random samples via permutation processes, and compare the observed statistic value to those of permutated samples. Some details about the permutation test can be referred to our previous work (Yuan et al., 2012). Here, we use the absolute difference of mean value between two samples as the statistic, $\text{s}=\left|\bar{X}-\bar{Y}\right|$. The *P*-value is calculated as the ratio of the number of permutated samples with statistical values larger than s to the total number of permutated samples.

The number of bases in the TD region between 2,000 and 10,000 is also estimated, and the detailed results are in Supplementary Text. From the comparison results, we can see that TARDIS has the highest F1-score when the purity is lower, followed by DINDT, TIDDIT, and DELLY. As purity increases, the F1-score of TIDDIT becomes the highest, and DINTD is slightly lower. When the purity is 0.9, the F1-score of the two is almost the same. From the comparisons of boundary bias, we can see that as the purity increases, the boundary bias decreases. When the tumor purity is relatively low, DELLY performs best. Although the average value of DINTD is similar to DELLY, there are some samples whose boundary bias deviates from the average value. When the tumor purity is increasing, the performance of DINTD improves, and the number of samples with large boundary bias is decreasing such that DINTD is slightly better than DELLY. Overall, the efficiency of DINTD is the best.

To demonstrate the efficiency of DINTD more comprehensively, we also performed experiments on all autosome chromosomes, and the detailed results are in Supplementary Text. In terms of sensitivity, precision, and F1-score, the results are similar to those of only chr21. In terms of boundary bias, the samples deviating from the average boundary bias decreased significantly.

### Application to the Real Samples

**FIGURE 5:**
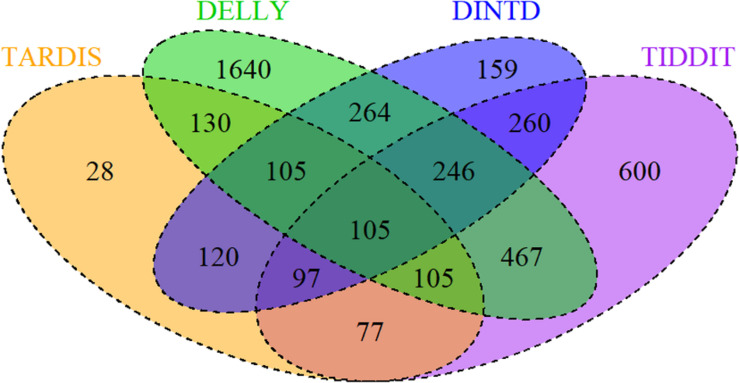
A Venn diagram for four samples (NA19238, NA19239, NA19240, HG00311) demonstrates the overlapping and non-overlapping TDs. The numbers here are the sum of TDs in the four samples. The orange, purple, green, and blue colors represent TARDIS, TIDDIT, DELLY, and DINTD, respectively.

To examine the effectiveness of DINTD, we further apply it to analyze four short sequencing samples from the 1000 Genomes project (Genomes Project et al., 2015; Sudmant et al., 2015). Three of the samples (NA19238, NA19239, NA19240) are from the Yoruba family trio. They are denoted as mother, father, and daughter, respectively. One of the samples (HG00311) is a Finnish male. All four are paired-end at 100bp for each read. DINTD is also applied to two ovarian cancer samples from EGA^3^. We perform the DINTD method and the other three methods on these samples. Due to the lack of ground truth about real data, we couldn’t calculate sensitivity, precision, F1-score, and boundary bias. To assess the methods and to provide a reliable measure, a Venn diagram is used to describe how these four methods are related. Figure 5 demonstrates the overlapping and non-overlapping TDs between each pair of methods for four samples from the 1000 Genomes project. From the Venn diagram where all the four samples are integrated, we can see that the DINTD method has a high relative consistency with the other methods.

The overlapping density score (ODS) (Yuan et al., 2018) is used to measure each method. The value of ODS for a method is calculated using the following formula:

(12) $$O\,D\,S=M_{o\,v\,e\,r\,l\,a\,p}\times \frac{M_{o\,v\,e\,r\,l\,a\,p}}{N_{c\,a\,l\,l\,e\,d}}.$$

Here, *M*_*overlap*_ represents the average of the number of overlaps of one method and the others. *N*_*called*_ represents the total number of TDs detected by the method. If the overlaps between different methods are assumed as true positives, then *M*_*overlap*_ can be assumed as sensitivity, and the ratio of *M*_*overlap*_ to *N*_*called*_ can be assumed as precision. ODS is somewhat similar to the area under the roc curve (AUC), and the higher the value of a method, the better the performance. The ODS calculation results of the four methods are shown in Table 1. We can see that DINTD has the highest ODS for the four samples, followed by TARDIS and TIDDIT, and then DELLY. So, we may conclude our proposed method is relatively reliable for real data applications.

**TABLE 1:** Comparison among the four methods in terms of ODS for four samples from 1000 Genomes project.

	TARDIS	TIDDIT	DELLY	DINTD
NA19238	55.37	48.49	42.46	57.49
NA19239	13.66	19.84	13.92	20.39
NA19240	17.71	26.71	20.04	29.03
HG00311	141.89	138.37	85.30	176.65

**FIGURE 6:**
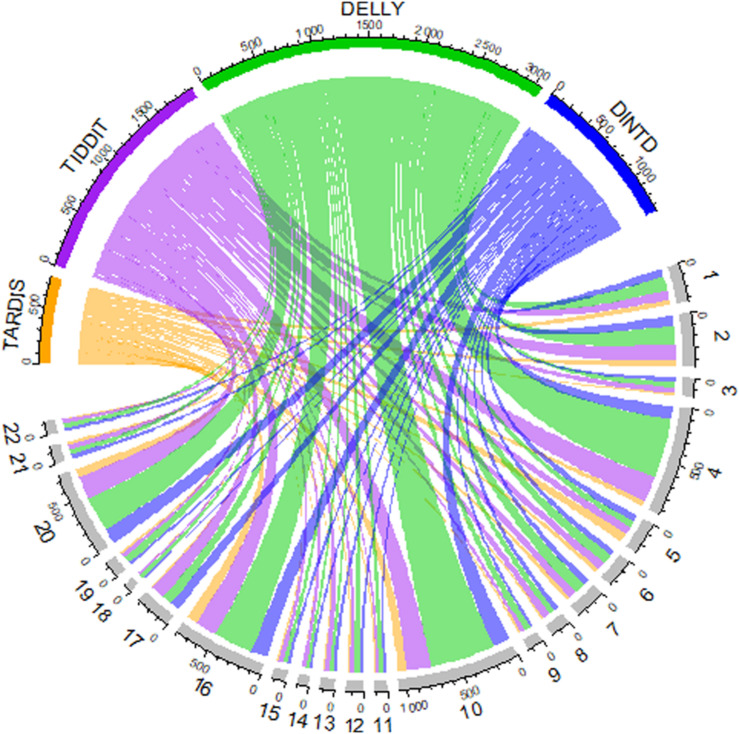
A Chord diagram demonstrates an overview of detected TD distribution for four samples (NA19238, NA19239, NA19240, HG00311). The orange, purple, green, and blue arcs in the upper half of the circle represent TARDIS, TIDDIT, DELLY, and DINTD, respectively. The gray arcs in the lower half of the circle represent autosome chromosomes.

We also show an overview of the detected TD distribution of the four methods in Figure 6. In the Chord diagram, the upper half of the circle is divided into four parts, and the color arcs orange, purple, green, and blue represent the method TARDIS, TIDDIT, DELLY, and DINTD, respectively. The lower half of the circle is divided into 22 parts, representing the autosome chromosomes from the 1st to 22nd. The widths of arcs of different colors from the upper half to the lower half represent the number of TDs found by a method on autosome chromosomes. The length of each arc in the upper half-circle represents the total number of TDs detected by this method, and the length of each arc in the lower half-circle represents the number of TDs detected on this chromosome. We find that the number of TDs detected by the TARDIS is the lowest, followed by DINTD, TIDDIT, and DELLY.

We further apply DINTD to two real ovarian cancer samples EGAR00001004796_2044_2 and EGAR00001004895_3705_2 from EGA. The results and detailed discussion are in Supplementary Text.

## Conclusion

We present a new method – DINTD – for the detection of TDs from short sequencing reads. It successfully builds a pipeline, the TD regions can be detected in the first stage using RD and MQ signals, and the regions are refined in the second stage using SR signals. Three new characteristics of DINTD can be summarized as: (1) The TD regions are detected using the DBSCAN algorithm and they are regarded as noise from clustering. To reduce the number of calculations, a strategy of the 2D binary search tree is embedded in DBSCAN to divide the search space; (2) To solve the problem of unsmoothed signals, the TV algorithm is used to denoise the RD and MQ signals; and (3) Through the analysis of the SR signals, the precise location of the TD region is inferred. However, if the clipping information is missing from the alignment records, DINTD cannot work. This kind of information is needed for the inference of the precise TD region boundary and is a limitation of DINTD.

The performance of DINTD is evaluated and validated through simulation tests and real sequencing data experiments. In the simulation tests, DINTD is compared with three other methods for sensitivity, precision, and F1- score. The boundary bias is also compared. In general, the results show that DINTD exhibits the best trade-off between sensitivity and precision, as well as for the boundary bias metrics. DINTD also is validated using several real sequencing samples and is compared with the other methods based on ODS. The results indicate that DINTD performs better than other methods. The computational complexity of DINTD is O(*m*+*nlogn*), where *m* is the number of reads in bam file and *n* is the number of bins. The detailed analysis is in Supplementary Text.

For future work, several points should be considered to improve the current DINTD. First, the detection of other mutations, such as CNV and interspersed TDs, should be analyzed. Second, to improve the efficiency of the variation detection algorithm, some intelligently-optimized clustering algorithms can be embedded in the current detection algorithm. Third, after the bin division, there are RD and MQ signals in each bin, resulting in too many values of RD and MQ signals. Whether the whole genome can be effectively divided according to the connection between bins should be explored.

## Data Availability Statement

The original contributions presented in the study are included in the article/Supplementary Material, further inquiries can be directed to the corresponding author.

## Author Contributions

JD and XY participated in the design of algorithms and experiments. JD, XY, and SW built the pipeline of rough TD detection and precise TD region inference. JD and MQ implemented the Python code. All authors read the final manuscript and agreed to the submission.

## Conflict of Interest

The authors declare that the research was conducted in the absence of any commercial or financial relationships that could be construed as a potential conflict of interest.

## Footnotes

1 https://ega-archive.org/

2 https://github.com/lh3/seqtk

3 https://ega-archive.org/.
