# Supplementary material for: DINTD: Detection and Inference of Tandem Duplications From Short Sequencing Reads
Source: Front Genet. 2020 Aug 11;11:924. doi: 10.3389/fgene.2020.00924 (PMC7433346; doi:10.3389/fgene.2020.00924)
Supplement: Supplementary file 1 [file Data_Sheet_1.docx]

# Influence of Parameters $\lambda$ and *len_bin* on Experiment Results

Different values of parameters *len_bin* and $\lambda$ will impact the results. The experiment results show that when *len_bin* is 2000 and $\lambda$ is 0.15, 0.35, and 0.45, respectively, the average performance of DINTD has not changed. But when *len_bin* is set to 1000, different $\lambda$ values will impact the experiment results. Taking Cov=10X and Tumor purity=0.3 as an example, the results of sensitivity, precision, and F1-score are summarized in Figure 1.1. We carefully observed and studied the cause of the performance degradation, and found that under other parameter settings, some bins with TDs were not detected by the DBSCAN algorithm in the first step of the algorithm core processing, which made the detected TD region inaccurate and the performance of algorithm decreased.


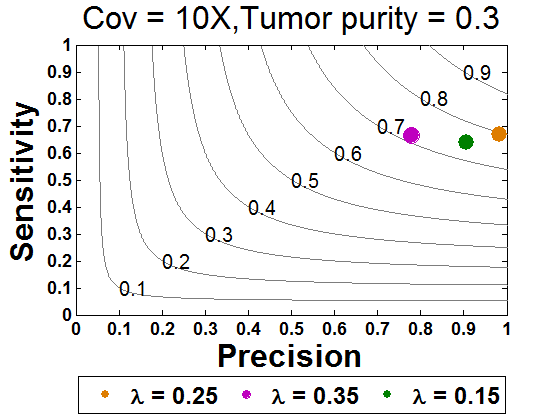


**Figure 1.1.** Sensitivity and precision with different values of $\lambda$ when the sequence coverage is 10X and tumor purity is 0.3. F1-score levels are compared and shown by the gray curves. Here, *len_bin* is set to 1000.

# Comparison of the Average Running Time


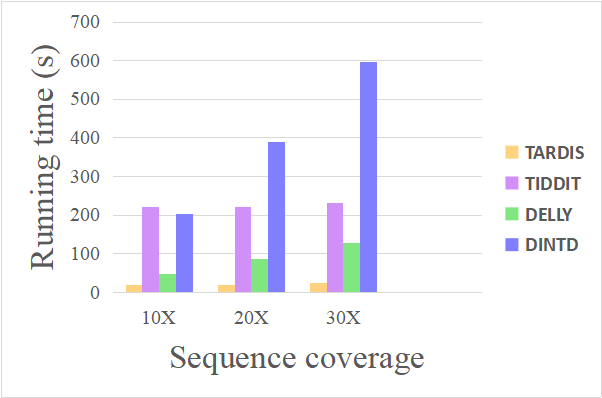


**Figure 2.1.** Comparison of the average running time of a sample. The dataset used is the simulated short sequencing dataset based on chromosome 21 in the reference hg19. The sequence coverage is 10X, 20X, and 30X, respectively. The tumor purity is set from 0.3 to 0.9. In each configuration, 10 TD regions are respectively embedded in 50 replicated samples and the number of duplications changes from 1 to 6. The number of bases in the TD region ranges from 10,000 to 50,000. The running times of the methods are related to the sequence coverage, so the result of comparison is demonstrated under the coverage of 10X, 20X, and 30X, respectively. Here, the unit of time (y-axis) is seconds. Overall, the running time of TARDIS is the shortest, followed by DELLY and TIDDIT, and the running time of DINTD is the longest.

# Comparisons of P-Values Between DINTD and Other Methods in Terms of the Significance of Boundary Bias Difference

**Table 3.1.** Cov=10X, Tumor purity=0.3

| Comparison between DINTD | P-value |
| --- | --- |
| DINTD - TARDIS | 0.1854815 |
| DINTD - TIDDIT | 0.00019998 |
| DINTD - DELLY | 9.999e-05 |

**Table 3.2.** Cov=10X, Tumor purity=0.4

| Comparison between DINTD | P-value |
| --- | --- |
| DINTD - TARDIS | 0.00019998 |
| DINTD - TIDDIT | 9.999e-05 |
| DINTD - DELLY | 9.999e-05 |

**Table 3.3.** Cov=20X, Tumor purity=0.3

| Comparison between DINTD | P-value |
| --- | --- |
| DINTD - TARDIS | 9.999e-05 |
| DINTD - TIDDIT | 0.05589441 |
| DINTD - DELLY | 0.00029997 |

**Table 3.4.** Cov=20X, Tumor purity=0.4

| Comparison between DINTD | P-value |
| --- | --- |
| DINTD - TARDIS | 9.999e-05 |
| DINTD - TIDDIT | 0.5243476 |
| DINTD - DELLY | 0.00519948 |

**Table 3.5.** Cov=30X, Tumor purity=0.3

| Comparison between DINTD | P-value |
| --- | --- |
| DINTD - TARDIS | 9.999e-05 |
| DINTD - TIDDIT | 9.999e-05 |
| DINTD - DELLY | 0.1136886 |

**Table 3.6.** Cov=30X, Tumor purity=0.4

| Comparison between DINTD | P-value |
| --- | --- |
| DINTD - TARDIS | 9.999e-05 |
| DINTD - TIDDIT | 9.999e-05 |
| DINTD - DELLY | 0.2540746 |

**Table 3.7.** Cov=30X, Tumor purity=0.7

| Comparison between DINTD | P-value |
| --- | --- |
| DINTD - TARDIS | 9.999e-05 |
| DINTD - TIDDIT | 9.999e-05 |
| DINTD - DELLY | 9.999e-05 |

# Comparisons of Experiment Results if the Number of Bases in the TD Regions is Between 2,000 to 10,000


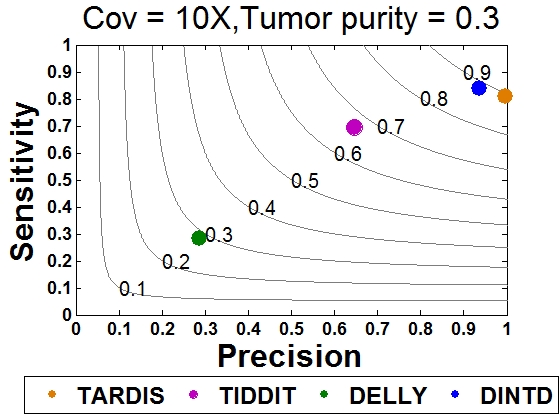

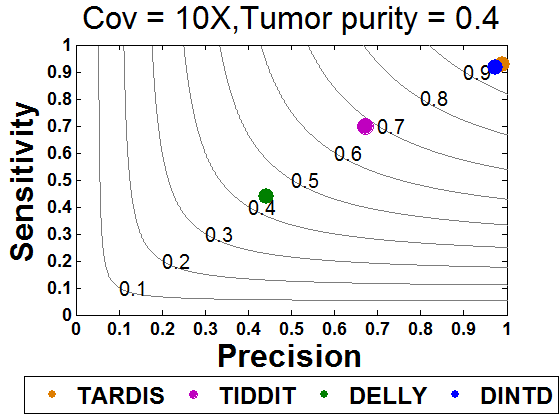


1. **(B)**


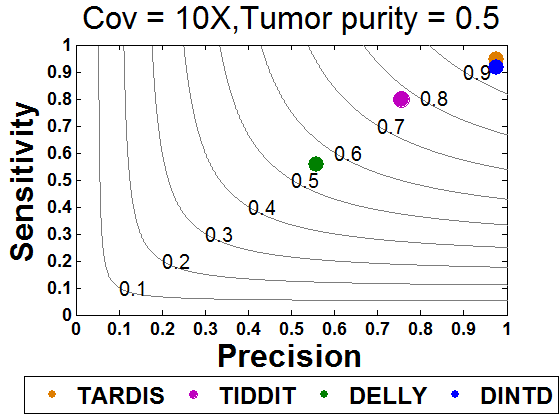

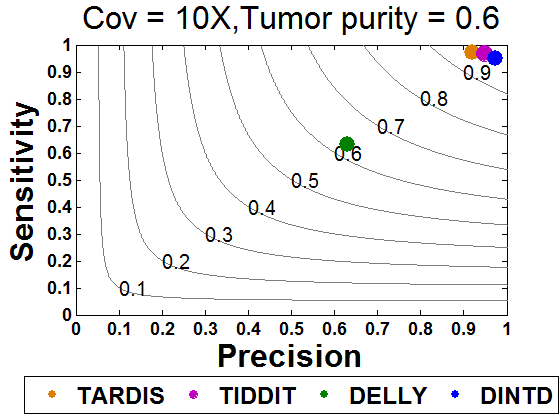


**(C) (D)**


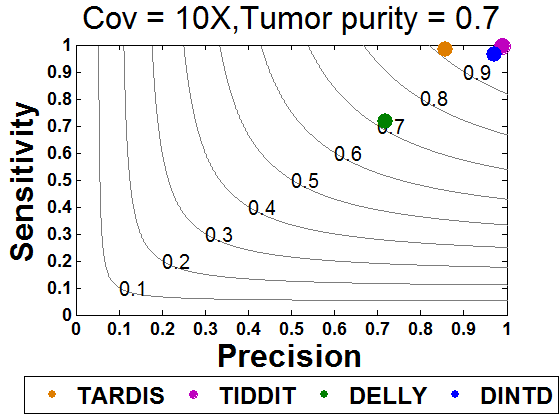

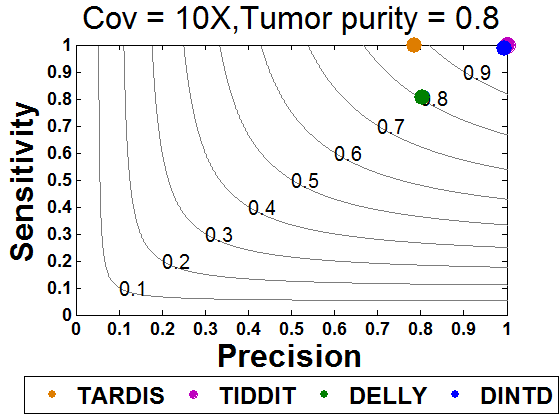


**(E) (F)**


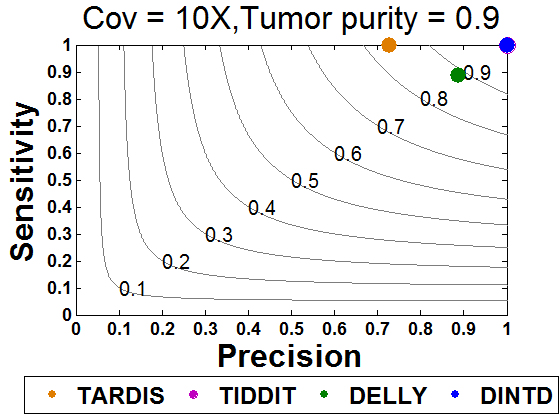


**(G)**

Figure 4.1. Sensitivity and precision between DINTD and three other methods (TARDIS, TIDDIT, and DELLY) when the sequence coverage is 10X. F1-score levels are compared and shown by the gray curves. Here, the number of bases in the TD region is between 2,000 to 10,000.


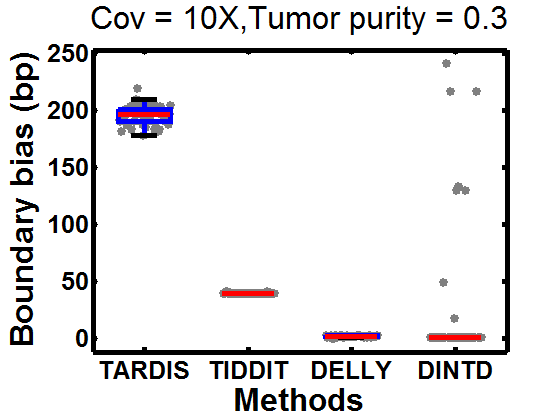

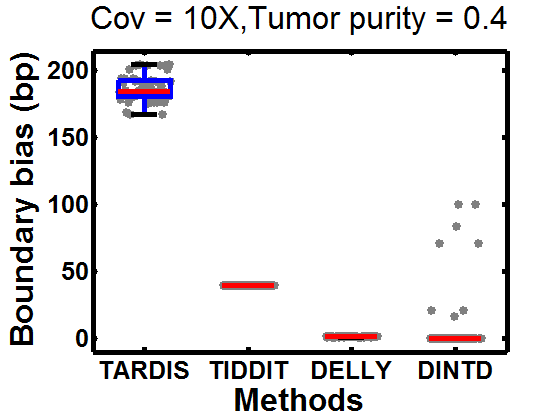


**(A) (B)**


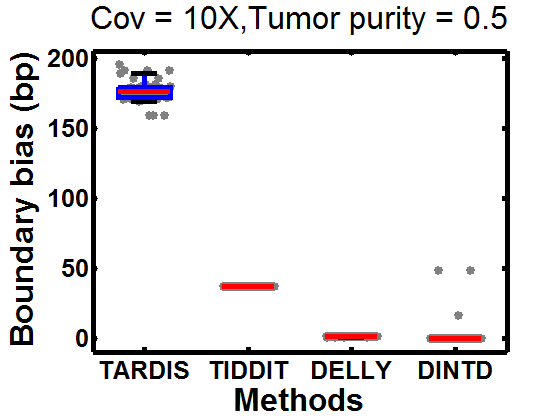

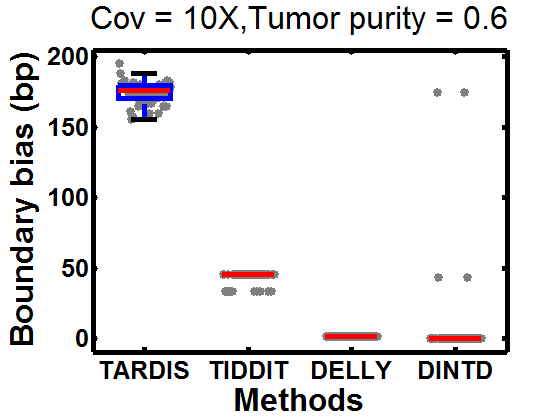


**(C) (D)**


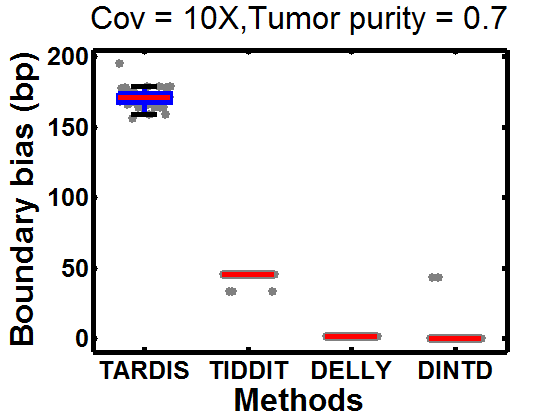

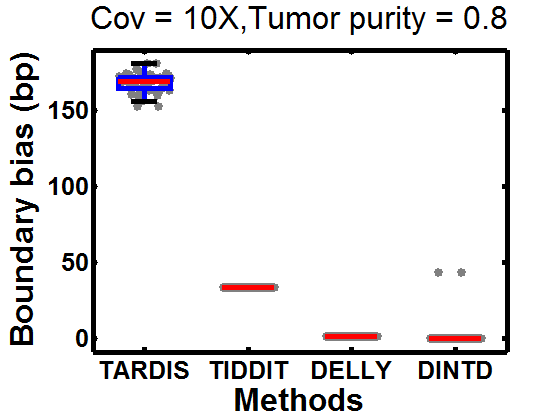


**(E) (F)**


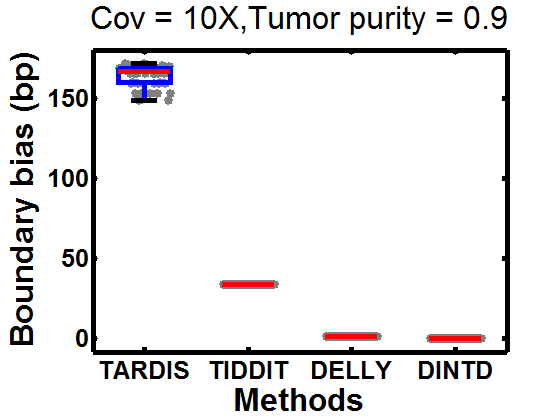


**(G)**

Figure 4.2. Comparisons of boxplot of the boundary bias between DINTD and three other methods (TARDIS, TIDDIT, and DELLY) when the sequence coverage is 10X. To better demonstrate the distribution of data, we draw boundary biases of 50 experiments under each configuration uniformly with gray dots under each method. Here, the number of bases in the TD region is between 2,000 to 10,000.

# Comparisons of Experiment Results on All Autosome Chromosomes


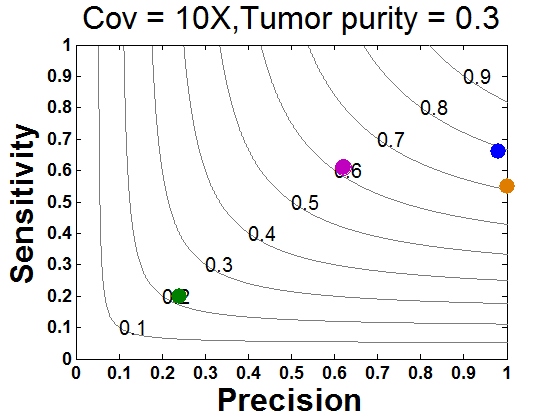

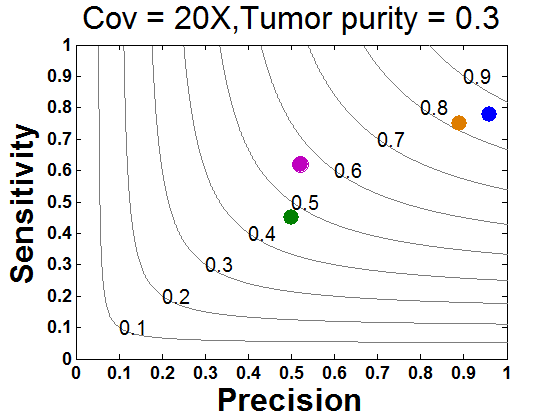

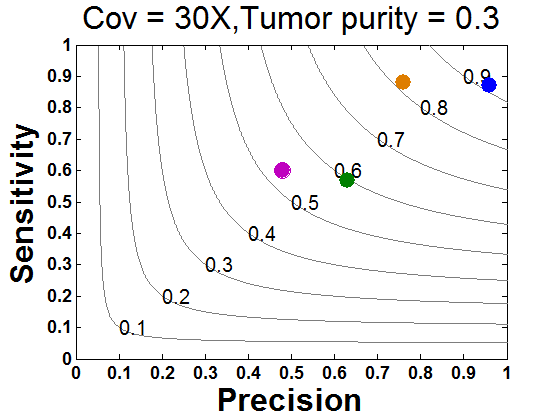


**(A) (B) (C)**


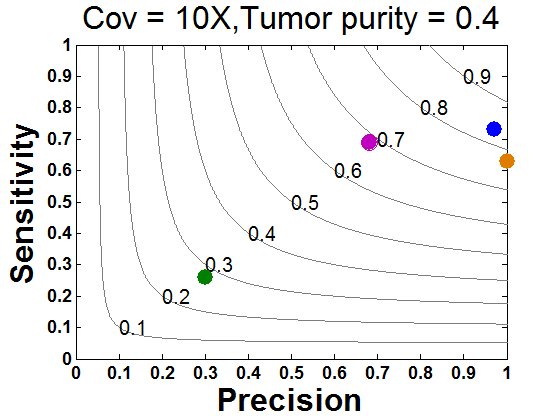

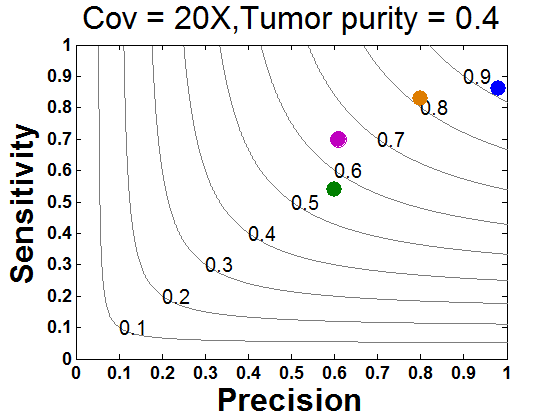

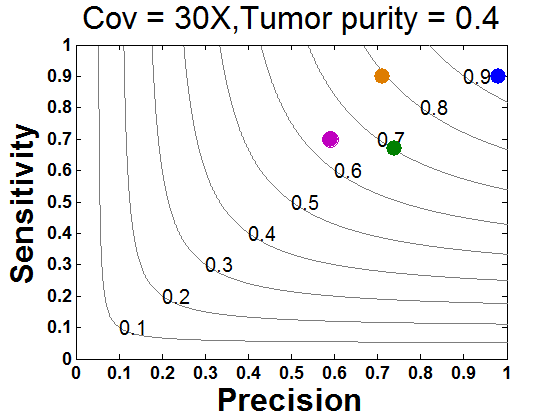


**(D) (E) (F)**


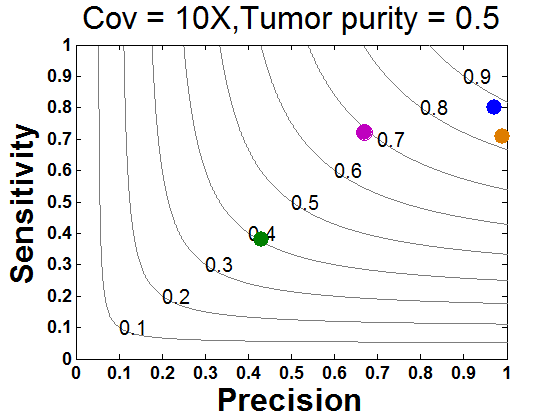

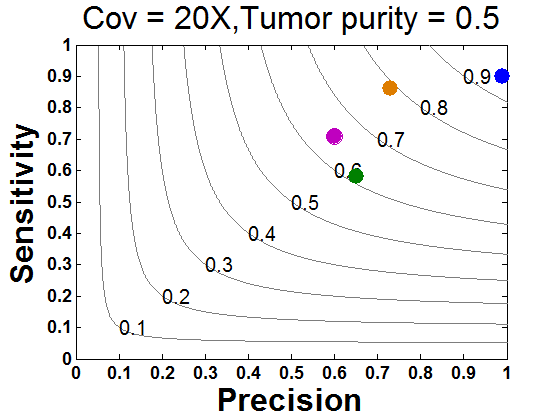

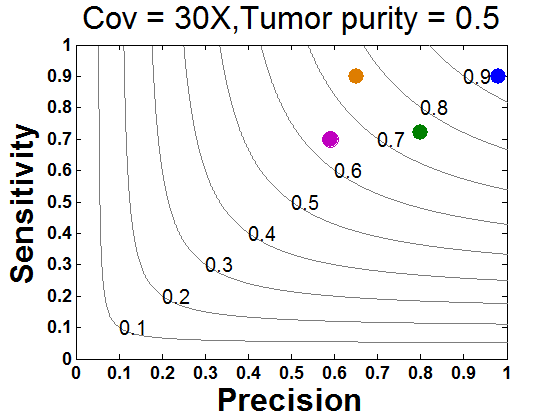


**(G) (H) (I)**


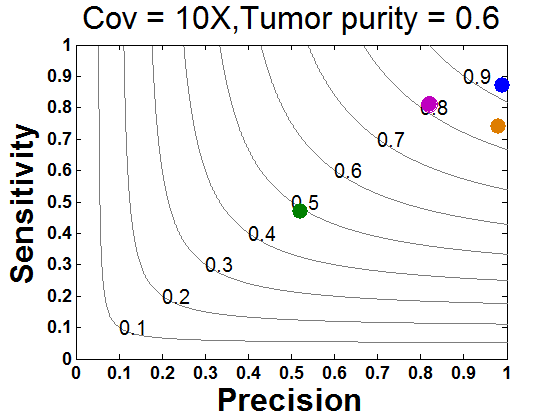

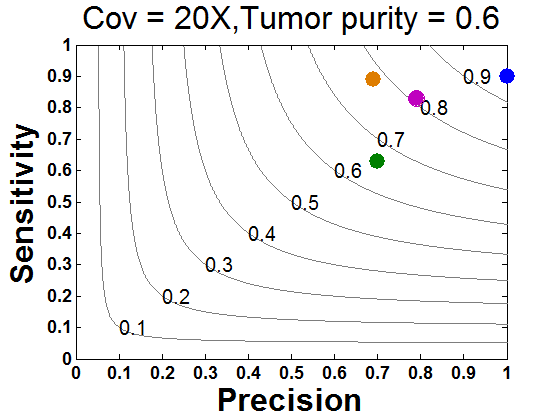

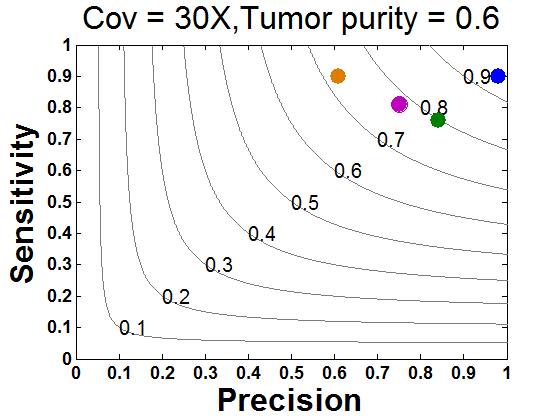


**(J) (K) (L)**


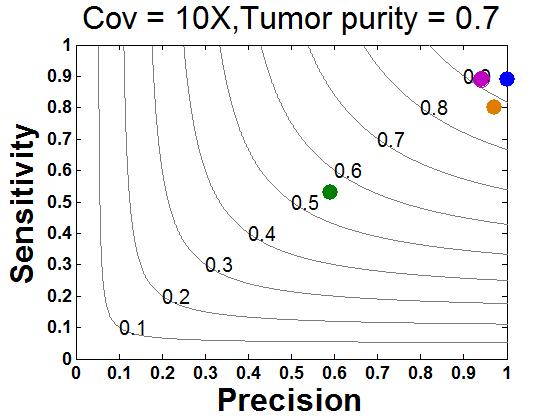

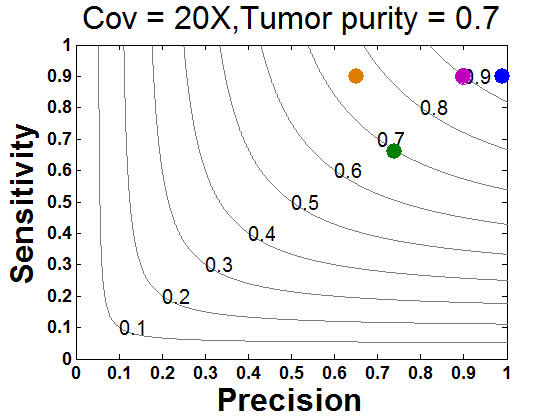

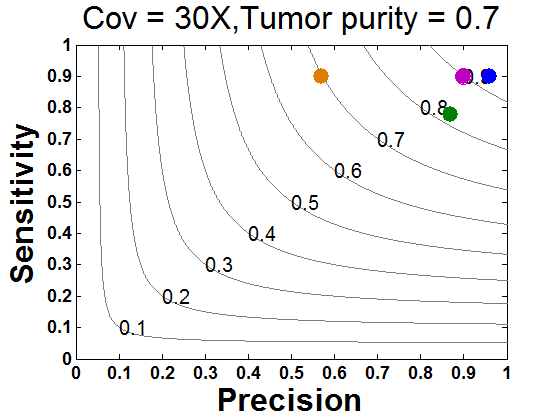


**(M) (N) (O)**


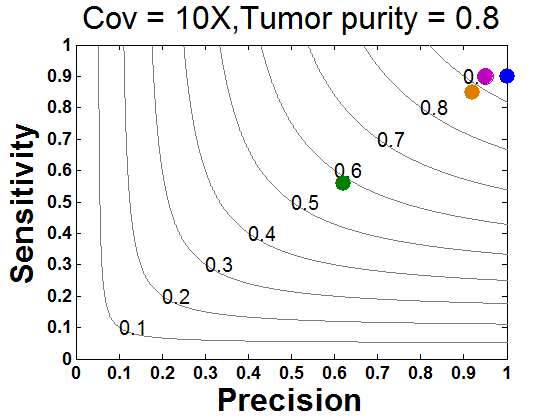

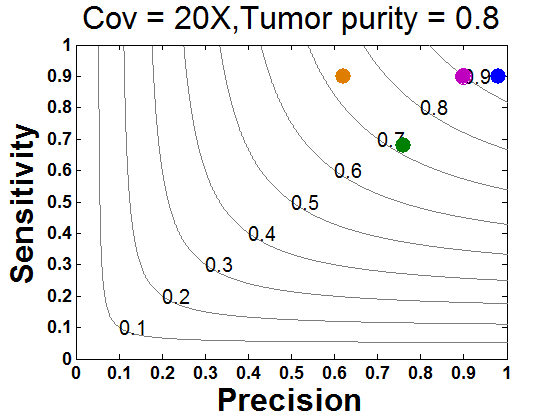

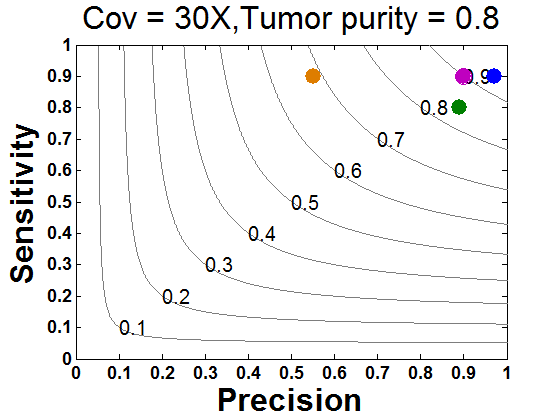


**(P) (Q) (R)**


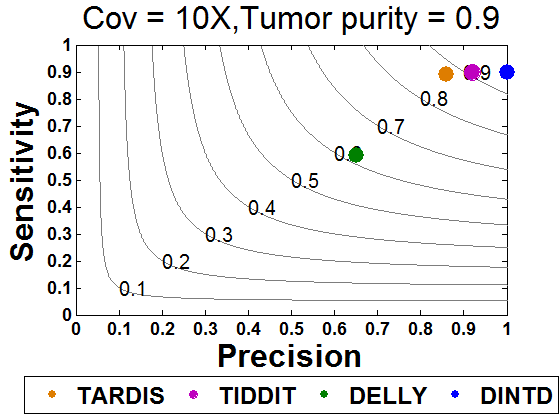

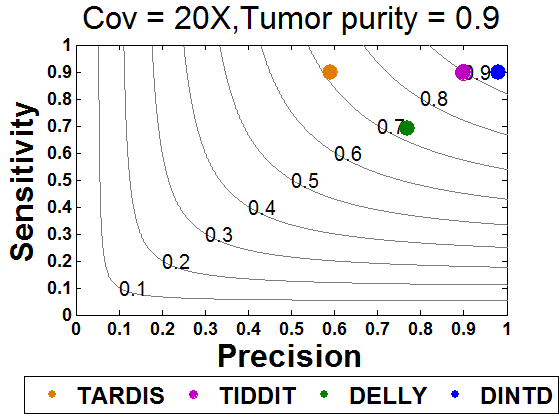

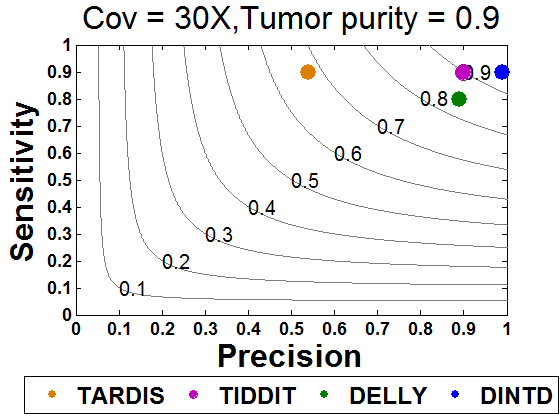


**(S) (T) (U)**

Figure 5.1. Comparisons of sensitivity and precision between DINTD and three other methods (TARDIS, TIDDIT, and DELLY) for all autosome chromosomes when the sequence coverage is 10X, 20X, and 30X. F1-score levels are compared as well and shown by the gray curves. The results for the whole genome are similar to that of only chr21.


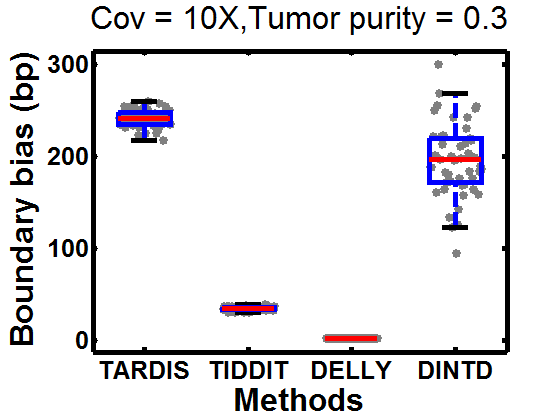

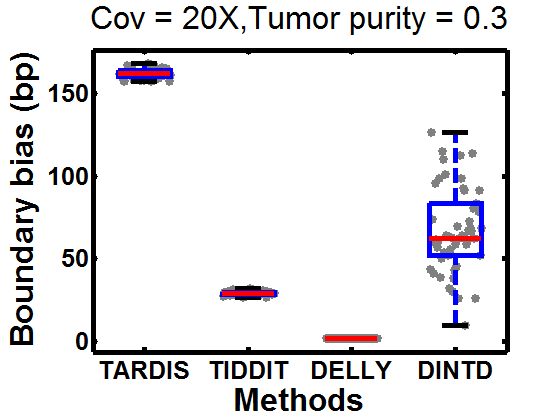

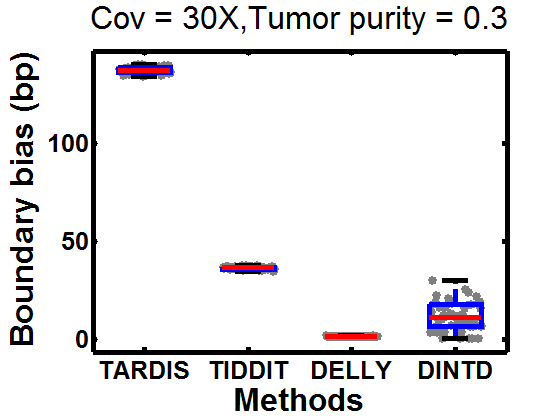


**(A) (B) (C)**


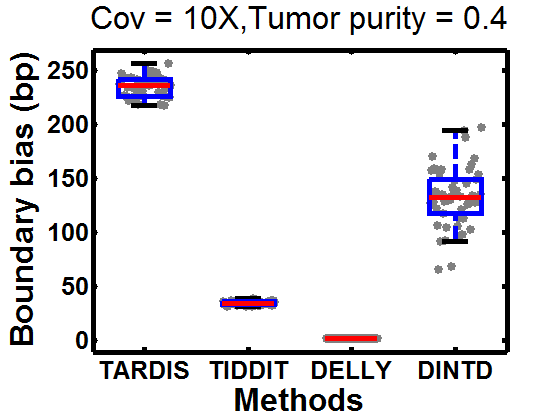

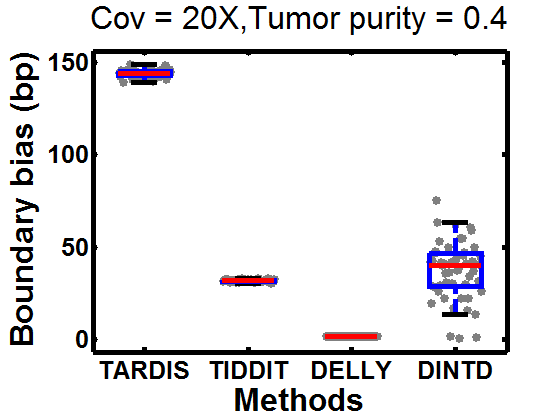

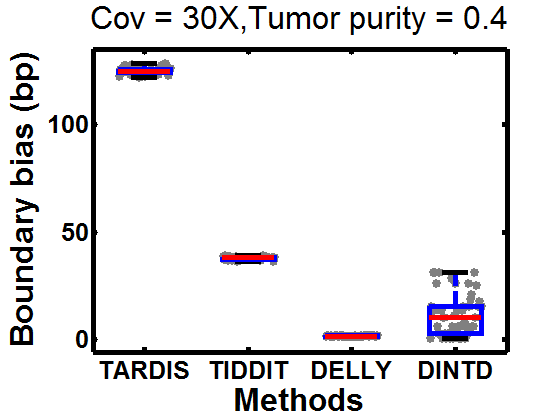


**(D) (E) (F)**


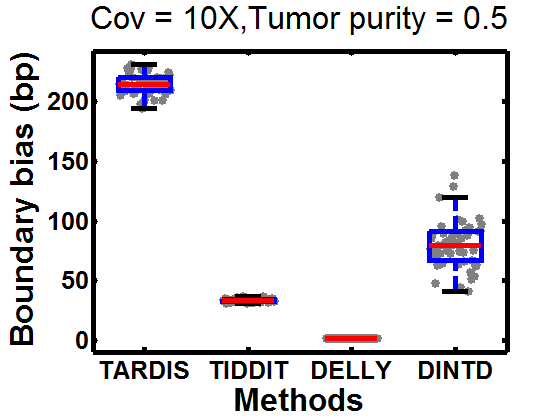

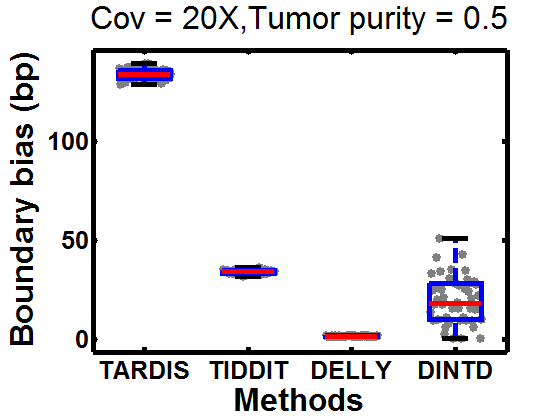

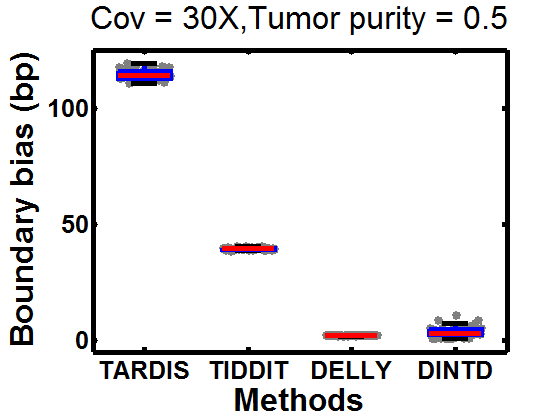


**(G) (H) (I)**


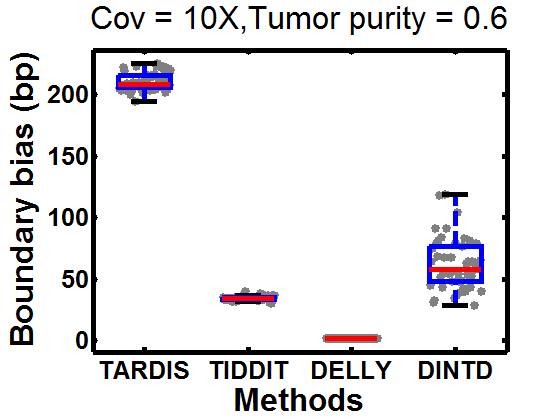

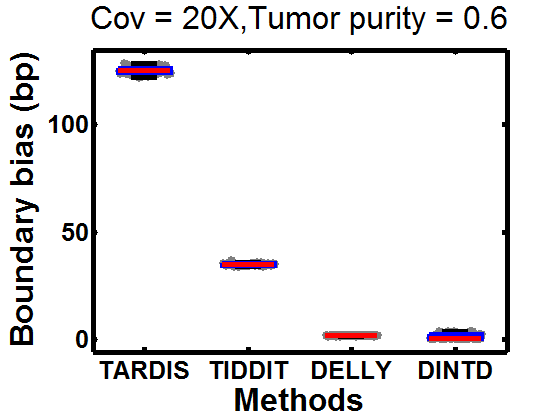

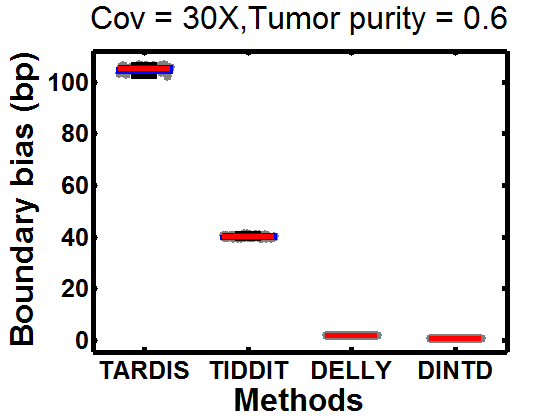


**(J) (K) (L)**


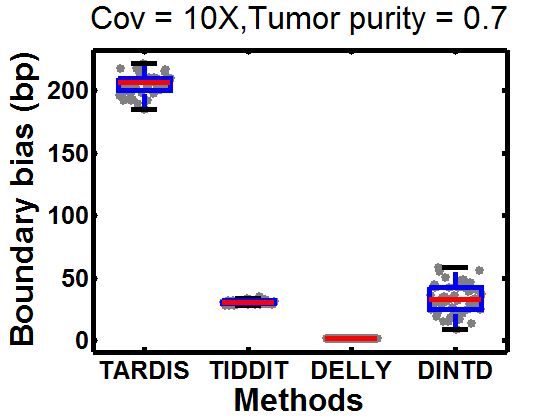

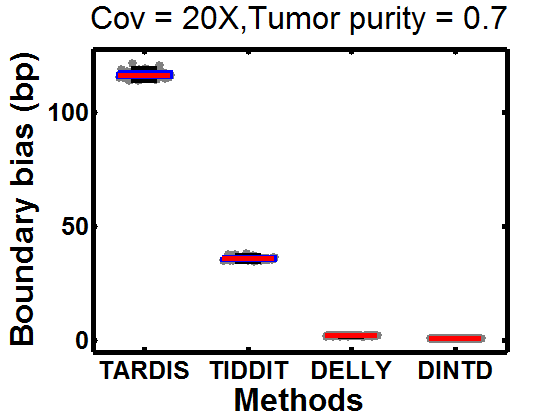

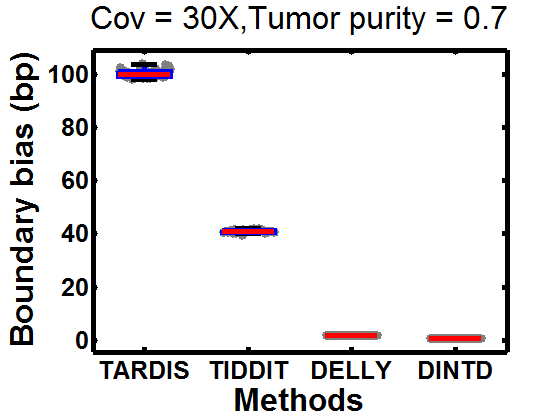


**(M) (N) (O)**


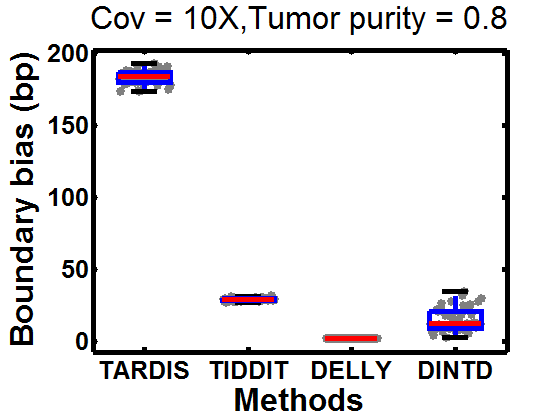

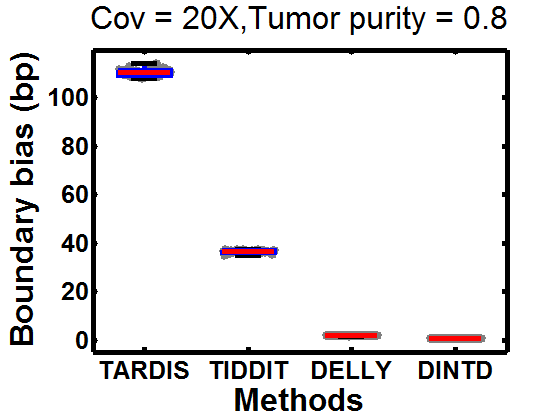

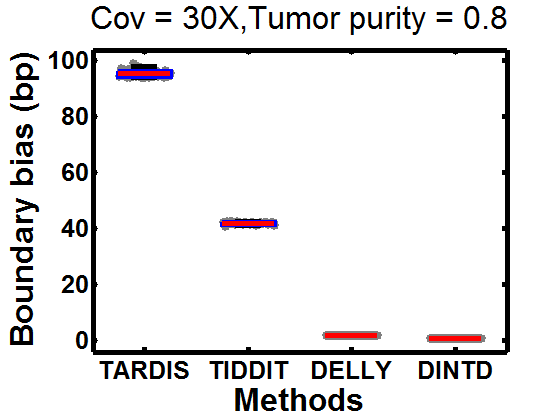


**(P) (Q) (R)**


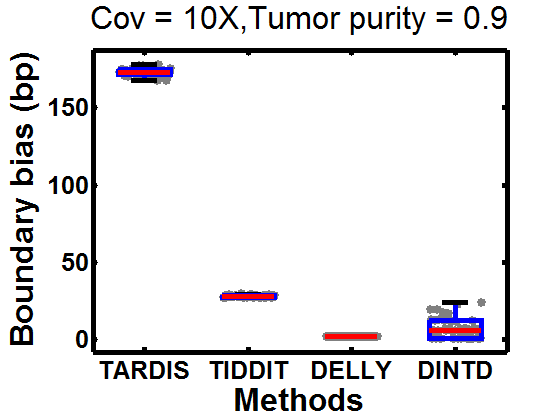

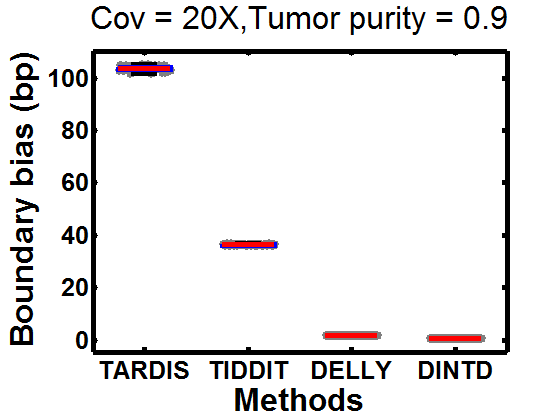

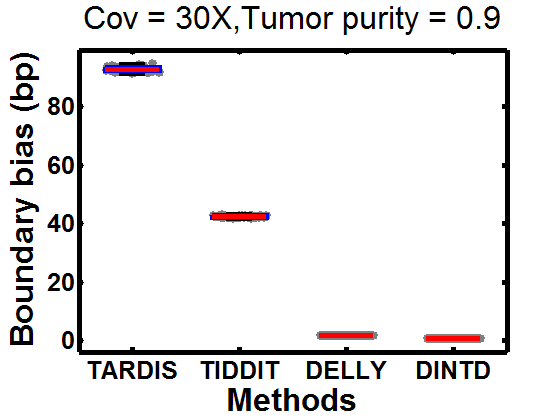


**(S) (T) (U)**

Figure 5.2. For all autosome chromosomes, comparisons of boxplot of the boundary bias between DINTD and three other methods (TARDIS, TIDDIT, and DELLY) when the sequence coverage is 10X, 20X, and 30X. To better demonstrate the distribution of data, we draw boundary biases of 50 experiments under each configuration uniformly with gray dots under each method. When compared with samples of only chr21, the results for all autosome chromosome genome are better, samples deviating from the average boundary bias decreased significantly.

# Applications to Two Real Ovarian Cancer Samples


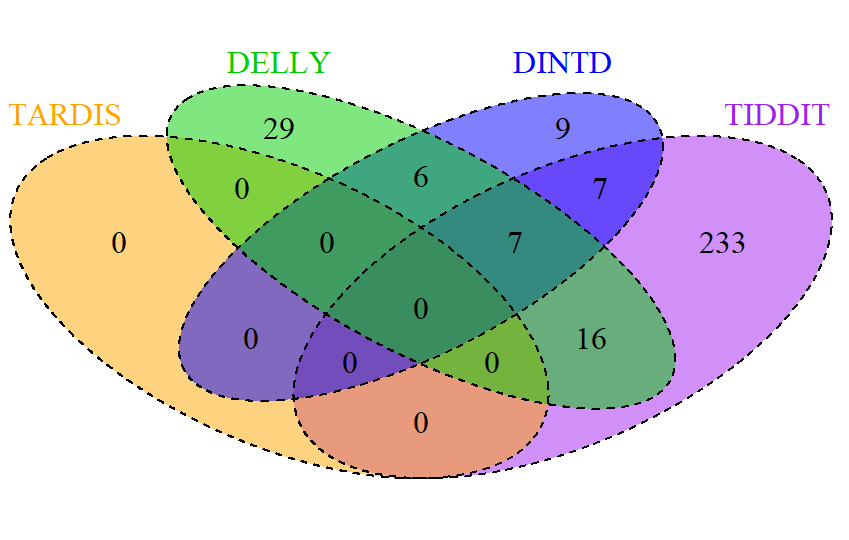


**Figure 6.1.** A Venn diagram demonstrates the overlapping and non-overlapping TDs for two real ovarian cancer samples EGAR00001004796_2044_2 and EGAR00001004895_3705_2 from EGA. The orange, purple, green, and blue colors represent TARDIS, TIDDIT, DELLY, and DINTD, respectively.

**Table 6.1.** Comparison of the three methods in terms of ODS. However, TARDIS did not find any TD, so its ODS value is not listed in the Table.

| Figure 1. | TIDDIT | DELLY | DINTD |
| --- | --- | --- | --- |
| EGAR00001004796_2044_2 | 0.32 | 0.96 | 1.17 |
| EGAR00001004895_3705_2 | 0.27 | 1.53 | 1.68 |


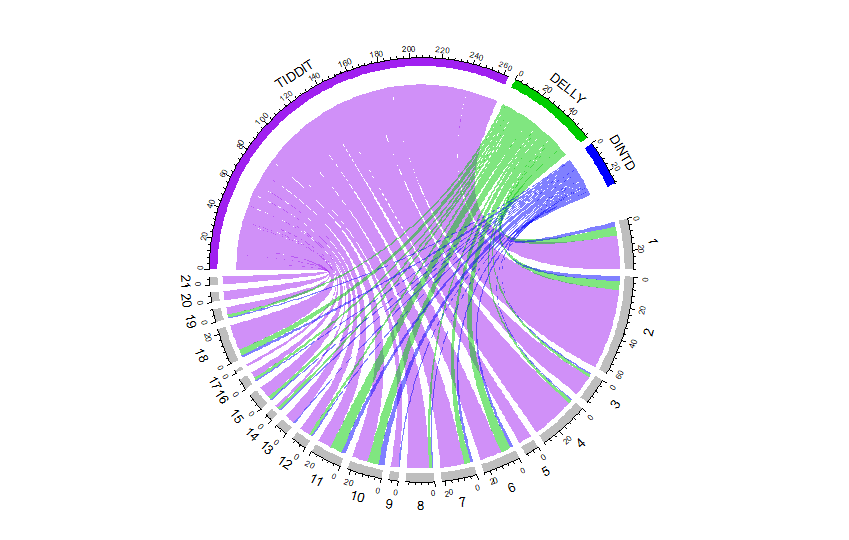
 **Figure 6.2.** A Chord diagram demonstrates an overview of detected TD distribution in the two real ovarian cancer samples. The purple, green, and blue arcs in the upper half of the circle represent TIDDIT, DELLY, and DINTD, respectively. The gray arcs in the lower half of the circle represent 22 autosome chromosomes.

# Analysis of Computational Complexity

The workflow of DINTD includes data preprocessing and core processes. In preprocessing of workflow, the calculation of read count and mapping quality has the most iterations, and the computational complexity is O(*m*), where *m* is the number of reads in the bam file. The core of the workflow is divided into two steps, detection of rough TDs and inference of precise TD regions. The second step is to find the exact boundary around each TD detected, and the first step is the most time-consuming. In the first step, DINTD uses DBSCAN algorithm to find the region where TDs may occur, then the time complexity is O(*n^2^*). At the same time, we use a 2D BST search strategy to improve the search of $\varepsilon$- neighborhood, so the computational complexity is O(*nlogn*), where *n* is the number of bins. Therefore, the overall computational complexity is O(*m*+*nlogn*).
